# Bacterial Manipulation of NK Cell Regulatory Activity Increases Susceptibility to *Listeria monocytogenes* Infection

**DOI:** 10.1371/journal.ppat.1005708

**Published:** 2016-06-13

**Authors:** Sarah E. Clark, Holly C. Filak, Brandon S. Guthrie, Rebecca L. Schmidt, Amanda Jamieson, Patricia Merkel, Vijaya Knight, Caroline M. Cole, David H. Raulet, Laurel L. Lenz

**Affiliations:** 1 Department of Biomedical Sciences, National Jewish Health, Denver, Colorado, United States of America; 2 Department of Immunology and Microbiology, University of Colorado School of Medicine, Aurora, Colorado, United States of America; 3 Department of Molecular and Cell Biology, Division of Immunology, University of California, Berkeley, Berkeley, California, United States of America; 4 Division of Pathology, Department of Medicine, National Jewish Health, Denver, Colorado, United States of America; 5 Department of Pediatrics, National Jewish Health, Denver, Colorado, United States of America; DUMC, UNITED STATES

## Abstract

Natural killer (NK) cells produce interferon (IFN)-γ and thus have been suggested to promote type I immunity during bacterial infections. Yet, *Listeria monocytogenes* (Lm) and some other pathogens encode proteins that cause increased NK cell activation. Here, we show that stimulation of NK cell activation increases susceptibility during Lm infection despite and independent from robust NK cell production of IFNγ. The increased susceptibility correlated with IL-10 production by responding NK cells. NK cells produced IL-10 as their IFNγ production waned and the Lm virulence protein p60 promoted induction of IL-10 production by mouse and human NK cells. NK cells consequently exerted regulatory effects to suppress accumulation and activation of inflammatory myeloid cells. Our results reveal new dimensions of the role played by NK cells during Lm infection and demonstrate the ability of this bacterial pathogen to exploit the induction of regulatory NK cell activity to increase host susceptibility.

## Author Summary

Natural killer (NK) cells are an innate immune cell population known to promote antiviral immunity through cytolysis and production of cytokines. Yet, some pathogens encode proteins that cause increased NK cell activation. Here, using a model of systemic infection by the bacterial pathogen *Listeria monocytogenes* (Lm), we show that NK cell activation increases host susceptibility. Activated NK cells increased bacterial burdens in infected tissues despite their early production of the pro-inflammatory cytokine IFNγ. We found that the ability of NK cells to exacerbate infection was independent from their production of IFNγ and instead due to subsequent production of the anti-inflammatory cytokine IL-10. A single bacterial protein, p60, was sufficient to elicit NK cell production of both early IFNγ and delayed IL-10. IL-10-production by NK cells has been shown to occur in other systems, but our studies are first to show how this “regulatory” response impacts the course of a bacterial infection. We found that IL-10 producing NK cells suppress accumulation and activation of inflammatory myeloid cells. Our studies suggest that the exploitation of NK cell regulatory activity provides selective pressure for the evolution of pathogen proteins that promote NK cell activation.

## Introduction

Immune defense against diverse pathogens requires timely recruitment of monocytes to sites of infection and activation of their antimicrobial functions [1]. IFNγ promotes antimicrobial activation of myeloid cells and is required for innate resistance to numerous pathogenic bacteria, including Lm [2,3]. Lm is an intracellular pathogen that causes severe and life-threatening infections primarily in elderly, pregnant, and immune compromised individuals [2–4]. In murine models of infection, Lm elicits a robust innate immune response that is characterized by IFNγ production by activated NK cells [5,6]. Memory-phenotype T cells can also serve as an early source of IFNγ following Lm and other bacterial infections [7,8]. NK cells protect against several viral infections and mediate anti-tumor immune responses in animal models and human patients [9,10]. Thus, NK cells have also often been assumed to confer protection during bacterial infections. However, there is a paucity of experimental evidence supporting a protective role for NK cells during *in vivo* antibacterial immune responses. Moreover, it has been puzzling that an Lm expressed virulence protein, p60, promotes NK cell activation and IFNγ production during infection [5,11].

NK cells were the first described innate lymphoid cell (ILC) population [12]. Activation of NK cell effector functions is regulated by germ line-encoded activating and inhibitory receptors [9]. Inhibitory NK cell receptors recognize host MHC or MHC-like molecules. Activating receptors recognize diverse stress-induced host proteins and, in some cases, microbe-encoded proteins [10]. Cytokines produced in response to infections also regulate NK cell activity. During Lm infection, the p60 protein appears to stimulate NK cell activation indirectly by promoting cytokine secretion from dendritic cells [11]. An abrupt increase in cytokines or activating receptor ligands or an encounter with target cells that have lost expression of ligands for inhibitory receptors licenses NK cells for cytolytic activity and secretion of IFNγ [9]. Some older studies provided evidence that depletion of NK1.1^+^ cells (which include both NK cells and NKT cells) increases host resistance to Lm infection [13,14]. The contributions of NK versus NKT cells to this phenotype and the mechanism for how these cells limit host resistance to Lm have not been described.

With appropriate stimulation, human and mouse NK cells have been observed to produce the immune regulatory cytokine IL-10 [15,16]. During Lm infection IL-10 has been shown to suppress both innate and adaptive immune responses and increases host susceptibility [17]. It is not known whether NK cells might be a crucial source of the IL-10 mediating these suppressive effects. However, one study provided evidence to suggest NK cells might produce IL-10 during Lm and *Toxoplasma* infections [18]. Additionally, IL-10 production by NK cells was shown to impair immunity during infection with the parasite *Leishmania* [19]. NK cells also have been shown to limit T cell responses during infections by MCMV and LCMV [20,21]. It is not known if or how NK cells affect adaptive immunity during Lm infection in wildtype mice. However, mice with a point mutation in NKp46 demonstrated hyper-activation of NK cells that correlated with reduced T cell responses to Lm-expressed ovalbumin [22]. These prior studies raised the hypothesis that NK cells responding to Lm infection might suppress host resistance through the production of IL-10, thus providing a rationale for Lm to express a protein that promotes NK cell activation.

Here, we used the murine model of systemic Lm infection to investigate how activation of NK cells and NK cell production of IFNγ impacts host susceptibility to this bacterial pathogen. Our results confirmed that NK cell activation exerts pro-bacterial effects. These effects were independent from IFNγ production and, in fact, NK cell IFNγ had no discernable effect on host resistance. Rather, we found that NK cells responding to Lm infection rapidly switched from IFNγ production to the secretion of IL-10. The secreted p60 virulence protein was sufficient to drive IL-10 production by mouse and human NK cells. IFNγ signaling in the NK cells dampened their IL-10 production. IL-10 producing NK cells were sufficient to dampen resistance to Lm infection and this regulatory activity was selectively associated with the suppression of inflammatory myeloid cell recruitment and activation. These data demonstrate the ability of a bacterial pathogen to exploit NK cell activation for selective suppression of innate immune responses during establishment of infection.

## Results

### NK cells increase susceptibility to Lm infection independent of IFNγ production

**Fig 1:**
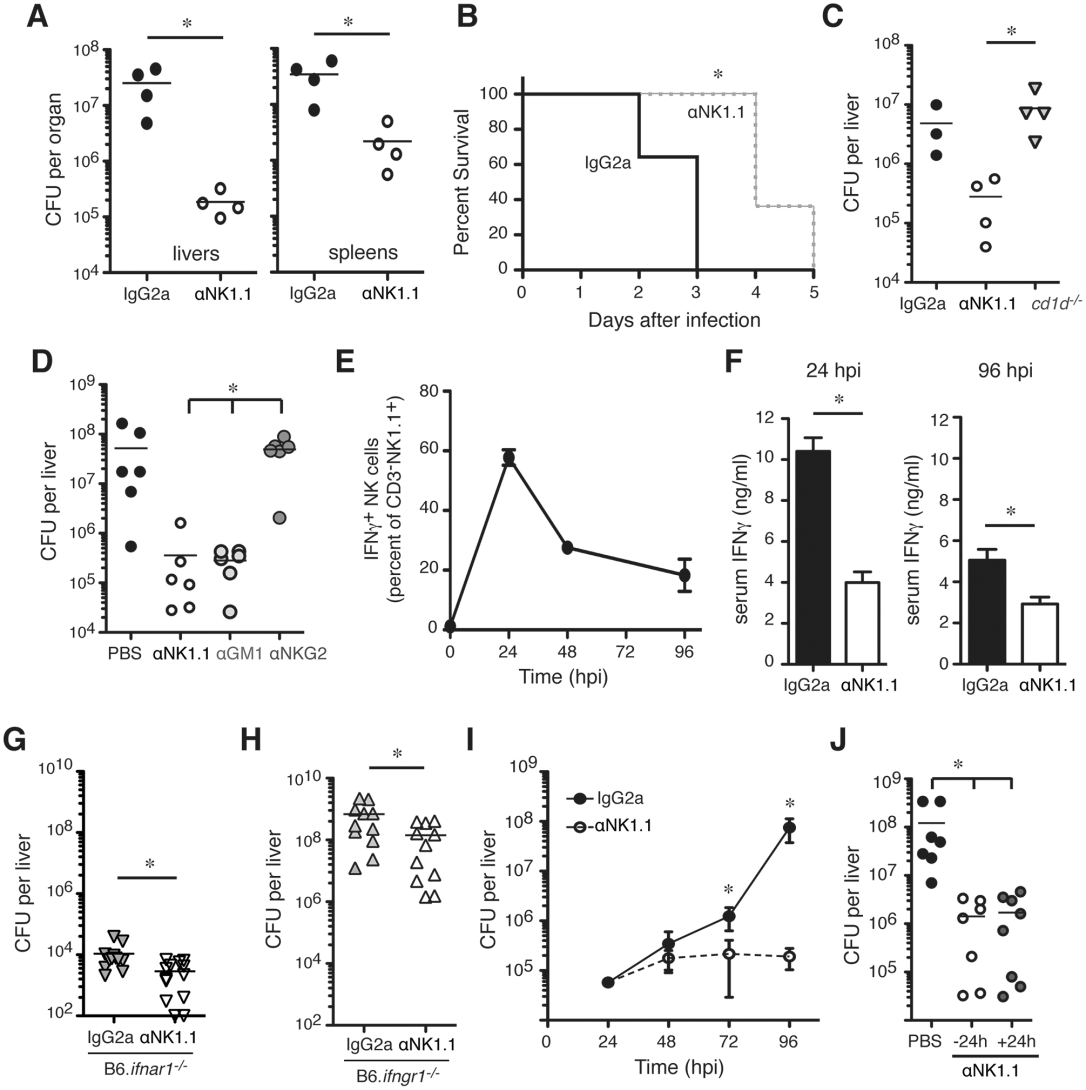
NK cells increase susceptibility to systemic *L*. *monocytogenes* (Lm) infection despite an independent from early IFNγ production. (**A**) Lm burdens from C57BL/6 (B6) mice treated with control (IgG2a) or NK1.1^+^ cell depleting (αNK1.1) antibodies (Abs) and infected 24 h later with 10^4^ Lm. Shown are total colony forming units (CFU) per tissue as determined by dilution plating of tissue lysates at 96 h post-infection (hpi). Symbols represent individual mice with mean (lines). Shown is one of five experiments using n = 3–5 mice/group. (**B**) Survival curve for B6 mice treated with IgG2a or αNK1.1 antibodies and infected 24 h later with 5 x 10^4^ Lm. Data are pooled from two experiments using n = 5–7 mice/group **(C)** Lm burdens from livers of B6 and NKT cell deficient (B6.*CD1d* ^-/-^) mice at 96 hpi. B6 mice were treated with IgG2a or αNK1.1 24 h before infection. Liver CFU from one of three experiments using n = 3–4 mice/group. (**D**) Lm burdens from livers of B6 mice treated 24 h before infection with αNK1.1^+^, anti-asialoGM1 (αGM1), or a non-depleting anti-NKG2A/C/E (αNKG2) Ab. Liver CFU at 96 hpi are shown for two pooled experiments representing n = 6 mice/group. (**E**) Proportions and total cell numbers of splenic NK1.1^+^CD3^-^ gated NK cells staining positive for intracellular IFNγ at the indicated hpi. Shown are mean ± SEM for data pooled from 3–5 experiments representing n = 9–18 mice/time point. (**F**) Serum IFNγ measured for control IgG2a or αNK1.1 treated B6 mice at 24 or 96 hpi. (**G**) B6.*ifnar1* ^-/-^ mice were treated with indicated Abs 24h before infection as above. Shown are liver bacterial burdens at 96 hpi. Bars depict mean ± SEM values from 3 pooled experiments using n = 3–5 mice/group. (**H**) B6.*ifngr1* ^-/-^ mice were treated with indicated Abs 24h before infection with 4 x 10^3^ CFU Lm. Livers were harvested for CFU counts at 72 hpi. Bars depict mean ± SEM values from 3 poled experiments using n = 3–5 mice/group. (**I**) B6 mice were treated with antibodies and infected as in (A). Liver bacterial burdens at 24, 48, 72, and 96 h after Lm infection. Data are mean ± SEM from 3–5 pooled experiments representing n = 9–18 mice/time point. (**J**) B6 mice were left untreated or administered αNK1.1 at 24 h before or 24 h after Lm infection. Data are pooled from two experiments with n = 3–4 mice/group. *P<0.05 as measured by (A, F-H) t-test or (B, D, I, J) ANOVA.

To investigate the impact of NK cells during bacterial infection, mice were depleted of NK1.1^+^ cells by a single injection of purified monoclonal Ab (αNK1.1) at 24 h prior to *i*.*v*. infection with 10^4^ live Lm (~0.5 LD_50_). This protocol eliminated splenic CD3^-^NK1.1^+^NKp46^+^ NK cells from the time of infection (0 h post infection; hpi) through 96 hpi (S1A Fig). At 96 hpi, Lm burdens in the depleted mice were observed to be 10–100 fold lower than in mice treated with an isotype control Ab (IgG2a) (Fig 1A). NK1.1 cell depletion was also effective at reducing bacterial burdens following low dose Lm infection (S1B Fig), and prolonged survival of mice infected with ~2.5 LD_50_ (Fig 1B). To address whether depletion of NK1.1^+^ NKT cells contributed to these effects, NKT cell-deficient B6.*cd1d* ^-/-^ mice were infected with Lm. Unlike αNK1.1 treatment, the absence of NKT cells in B6.*cd1d*-/- mice had no significant effect on Lm burdens (Fig 1C). Antisera specific for the ganglioside asialo-GM1 (αGM1) also depletes NK cells but does not deplete conventional NKT cells [23]. Lm burdens in B6 mice treated with αGM1 before infection were identical to those in mice treated with αNK1.1 (Fig 1D). Depleting just the subset of NK cells expressing Ly49C/I also significantly reduced Lm burdens, though not to the extent as seen with αNK1.1 (S1C Fig). However, treatment of mice with a non-depleting monoclonal Ab that binds the NK cell surface markers NKG2A/C/E (αNKG2) did not impact Lm burdens (Fig 1D). These data indicated that removing NKT cells had no effect on Lm burdens whereas depletion of NK cells or a subset of NK cells dramatically suppressed bacterial survival and growth in host tissues. We conclude that the presence of NK cells acts to increase host susceptibility to Lm and that the protective effects of αNK1.1 treatment are due to depletion of NK cells.

NK cells were the largest population staining positive for intracellular IFNγ at 24 hpi (S1D and S1E Fig), which corresponded to the peak of their IFNγ production as determined by intracellular staining (Fig 1E). Serum IFNγ concentrations were reduced significantly in mice depleted of NK1.1^+^ cells, particularly at 24 hpi (Fig 1F). T cells also stained positive for intracellular IFNγ and were likely the source of residual serum IFNγ in the depleted mice (S1D and S1F Fig). We have previously shown that production of type I interferon down-regulates IFNGR, reducing host resistance to Lm [24]. To evaluate whether the effects of αNK1.1 treatment were dependent on type I interferon or IFNγ signaling, we evaluated Lm burdens in mice lacking expression of the type I interferon receptor (B6.*ifnar1* ^-/-^) or the IFNγ receptor (B6.*ifngr1* ^-/-^). Despite the extreme differences in susceptibility of these mouse strains to Lm, depletion of NK1.1^+^ cells was protective in both (Fig 1G and 1H). We also found that NK cell depletion did not impact Lm burdens until 72 hpi (Fig 1I), well after the peak of IFNγ production by NK cells (Fig 1E). Finally, when early NK cell IFNγ production was allowed to occur prior to αNK1.1 treatment, NK1.1^+^ cell depletion remained highly effective at reducing Lm burdens (Fig 1J). These results indicated that in mice with an intact T cell compartment NK cell production of IFNγ has no discernable impact on host resistance or susceptibility to Lm, arguing the pro-bacterial effects of NK cells are not due to hyper-production of IFNγ.

### Lm infection stimulates NK cell production of IL-10

**Fig 2:**
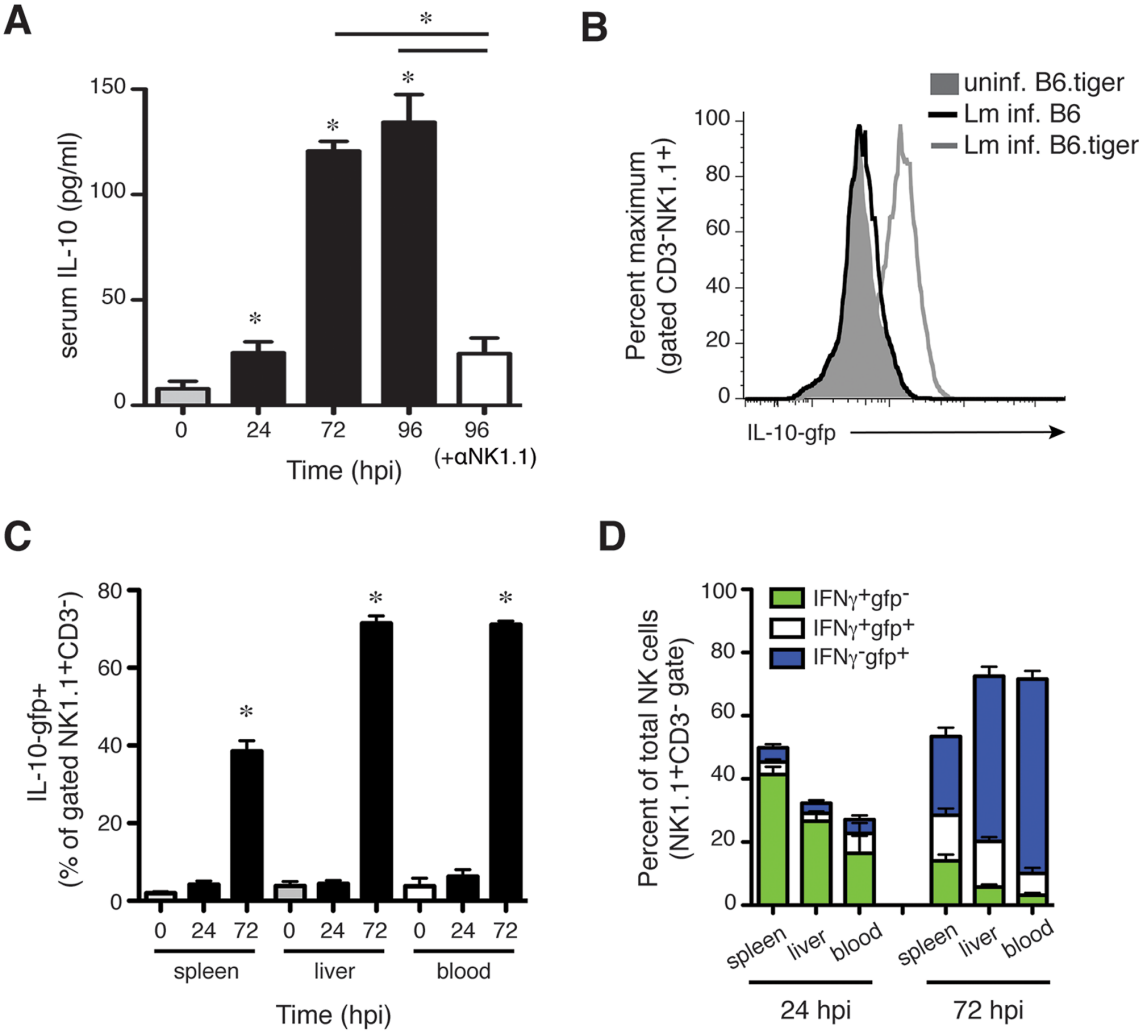
NK cells responding to Lm infection acquire the ability to produce IL-10. (**A**) Serum IL-10 concentrations at the indicated times after Lm infection of control IgG2a or αNK1.1 treated B6 mice. Data are pooled from 3 experiments for n = 9–12 mice/group. (**B**) Histogram depicting the staining for IL-10-gfp reporter in gated CD3^-^NK1.1^+^ NK cells from livers of uninfected IL-10-gfp+ (B6.tiger) mice or B6.tiger and control B6 mice at 72 hpi with Lm. (**C**) Shown are mean ± SEM percentages of gated CD3^-^NK1.1^+^ cells that stained positive for IL-10-gfp prior to infection or at 24 or 72 hpi with Lm. Gated NK cells from spleen, liver, and blood of B6.tiger mice were analyzed. Data are pooled from 3 experiments and represent n = 9–14 mice per time point. **(D)** Proportions of IFNγ^+^ and IL-10^+^ populations of NK1.1^+^CD3^-^ gated NK cells from the indicated tissues at 24 or 96 hpi. Data are pooled from three experiments with n = 3–5 mice/group. Pooled from three experiments representing n = 9–12 mice/group. *P< 0.05 as determined by (A, C) ANOVA.

NK cells were previously shown to have the capacity to produce IL-10 at late stages of infections by the parasites *Leishmania donovani* and *Toxoplasma gondii* and viruses such as murine cytomegalovirus (MCMV) [18,19,25]. In one of these studies, IL-10-gfp^+^ NK cells were also observed at 4 dpi with Lm in Vert-X IL-10 GFP-reporter mouse [18]. We thus speculated that NK cells might be a source of IL-10 during Lm infection. Consistent with this hypothesis, we observed elevated serum IL-10 concentrations by 72–96 hpi in control but not αNK1.1-treated mice (Fig 2A). To more directly assess the potential for IL-10 production by NK cells, we evaluated gfp staining in NK cells from tiger IL-10 GFP-reporter mice infected with Lm [26]. In tiger IL-10 GFP-reporter mice the 3’UTR of IL-10 remains unaltered, unlike Vert-X and other IL-10 GFP-reporter mouse strains that contain a mRNA-stabilizing sequence [27]. This preserves post-transcriptional regulation of the labile il10 transcripts and thus permits more reliable detection of IL-10-producing cells over time without ex-vivo re-stimulation. At 72 hpi, gfp staining was selectively increased in NK cells from spleens, livers, and blood of Lm-infected reporter mice versus uninfected reporter mice or Lm-infected control B6 mice (Fig 2B and 2C). IL-10-gfp reporter expression was not observed at 24 hpi (Fig 2C). Intracellular staining for IFNγ confirmed the differing kinetics of IFNγ and IL-10-gfp production and demonstrated that only a small population of NK cells produced both cytokines (Fig 2D and S2 Fig). Thus, the timing of NK cell IL-10-gfp reporter activity correlated well with NK cell-dependent increases in serum IL-10 (Fig 2A) and Lm burdens (see Fig 1I). These data further suggested that most NK cells responding to Lm infection are committed to either IFNγ or IL-10 production at these time points during the infection.

### The Lm p60 virulence protein promotes NK cell IL-10 production

**Fig 3:**
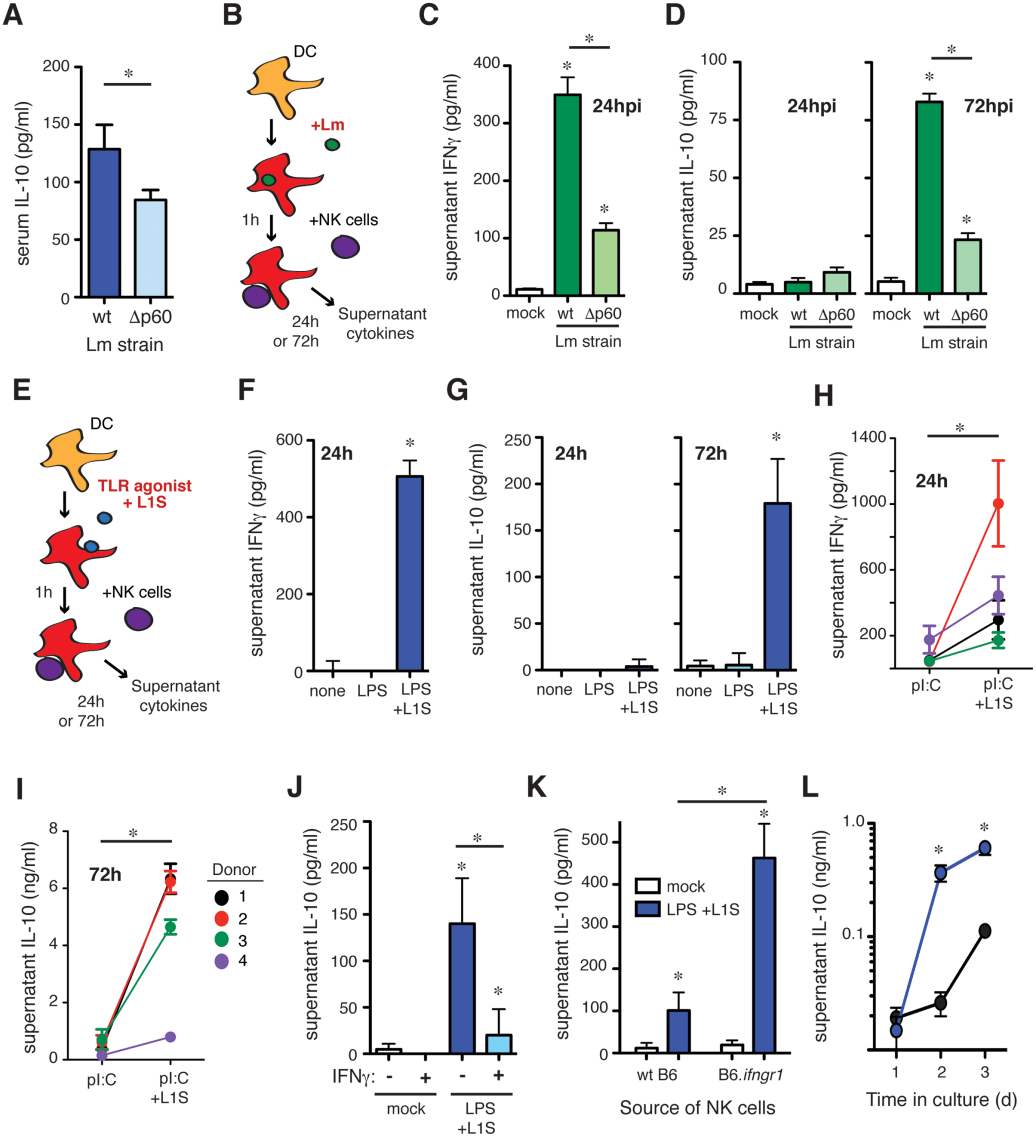
The L1S region of the Lm p60 protein is sufficient to induce NK cell IL-10 production. **(A)** Serum IL-10 at 96 hpi in B6 mice infected with wt or p60-deficient (Δp60) Lm strains. **(B)** Schematic of co-culture system consisting of BMDC infected with Lm for one hour, followed by washing and the addition of splenic purified NK cells at 2 h post-infection. **(C)** 24 h supernatant IFNγ after infection of BMDC with wt or p60-deficient Lm in co-culture with wt NK cells. **(D)** 24 h and 72 h supernatant IL-10 after infection of IL-10 deficient BMDC with wt or p60-deficient Lm in co-culture with wt NK cells. **(E)** Schematic of co-culture system consisting of BMDC treated with a TLR agonist ± L1S for one hour, followed by washing and the addition of splenic purified NK cells at 2 h post-stimulation. **(F)** 24 h supernatant IFNγ after treatment of BMDC with LPS ± L1S in co-culture with wt NK cells. **(G)** 24 h and 72 h supernatant IL-10 after treatment of IL-10 deficient BMDC with LPS ± L1S in co-culture with wt NK cells. **(H)** 24 h supernatant IFNγ after treatment of human DC with polyI:C ± L1S in co-culture with PBMC from unrelated donors. **(I)** 72 h supernatant IL-10 after treatment of human DC with polyI:C ± L1S in co-culture with PBMC from unrelated donors. **(J)** 72 h supernatant IL-10 after treatment of IL-10 deficient BMDC with LPS + L1S ± stimulation with 50 ng/mL IFNγ in co-culture with wt NK cells. **(K)** 72 h supernatant IL-10 after stimulation of IL-10 deficient BMDC with LPS + L1S in co-culture with NK cells from wt or IFNGR^-/-^ mice. (**L**) IL-10 was measured in supernatants collected at 24, 48, and 72 h from L1S-stimuated co-cultures of IL-10 deficient BMDC and NK cells from wt or IFNGR^-/-^ mice. Data for (C, D, F, G, H, I, J, K, L) pooled from three or more experiments. *P<0.05 as determined by t-test.

We previously reported that the N-terminal LysM domain (LysM1) of the secreted Lm virulence protein p60 indirectly promotes NK cell IFNγ secretion during systemic infection and in cell culture studies. This protein domain stimulates DC production of cytokines including IL-12 and IL-18 that together with cell-contact stimulate NK cell IFNγ production [5,11]. Given the protective effects of IFNγ, it has not been clear how the pathogen might benefit from stimulating these responses. We thus asked whether Lm expression of p60 might also promote NK cell IL-10 secretion. Consistent with this hypothesis, serum IL-10 was significantly reduced in mice infected with an Lm strain deficient in p60 (Δp60; Fig 3A). However, the Δp60 strain is also attenuated in vivo, though this does not impair Lm infection of cultured macrophages or DCs [11,28]. To more directly investigate whether p60 expression permits Lm to stimulate NK cell IL-10 production we used a co-culture system consisting of bone marrow-derived DC (BMDC) and purified splenic NK cells [29]. Use of IL-10-deficient BMDC ensured any IL-10 in these cultures was derived from NK cells. BMDC were infected with wt Lm or the Δp60 strain. At 1hpi the BMDC were washed and media containing gentamicin was added. Purified splenic NK cells were added at 2 hpi (Fig 3B). Under these conditions, any IFNγ produced in the cultures is dependent on NK cells ([11,29]). Consistent with our prior findings, BMDCs infected with Δp60 Lm elicited significantly less NK cell-dependent IFNγ than those infected with wt Lm (Fig 3C). As shown above, NK cell IFNγ production during systemic Lm infection peaks at 24 hpi (Fig 1E and 1F), while IL-10 production peaks later (Fig 2A). Consistent with these results, IL-10 was not detected in culture supernatants at 24 hpi, but was reproducibly detected by 72 hpi (Fig 3D). As seen in serum, IL-10 concentrations in culture supernatants were also significantly reduced following infection with Δp60 Lm. These data suggested Lm expression of p60 stimulates DC to promote serial NK cell secretion of IFNγ and IL-10.

To specifically investigate whether the region of p60 protein that stimulates NK cell IFNγ secretion also promotes secretion of IL-10, BMDC were primed with TLR agonists and stimulated with a recombinant p60 fragment (L1S) that contains the LysM1 domain (Fig 3E). As previously reported [11], IFNγ secretion was observed selectively in 24 h co-cultures where BMDC and NK cells were stimulated with a priming agent (LPS) and L1S protein (Fig 3F). L1S also induced NK cell IL-10 secretion, but again this was selectively observed in the 72 h cultures (Fig 3G). As for NK cell production of IFNγ, IL-10 production required stimulation with both TLR agonist and L1S protein. Thus, our data suggested L1S stimulates primed BMDCs to promote NK cell activation for sequential IFNγ and IL-10 production. To determine whether human NK populations were also responsive to L1S, DCs were cultured 7 days from healthy donor PBMCs, then stimulated with a priming agent (pI:C) ± L1S and purified autologous NK cells as in Fig 3E. The results using human cells paralleled those above. Co-cultures with cells from 4/4 donors produced IFNγ at 24 h (Fig 3H) and IL-10 at 72 h (Fig 3I) in response to the L1S treatment. Thus, the L1S fragment of the Lm p60 protein is necessary and sufficient to stimulate the ability of primed DCs to promote early IFNγ and delayed IL-10 secretion from both murine and human NK cells.

### IFNγ signaling suppresses NK cell production of IL-10

Because IL-10 production by both mouse and human NK cell cultures was delayed relative to IFNγ production we considered whether production of IFNγ might inhibit NK cell IL-10 secretion. Subsequent to L1S or control stimulation, recombinant IFNγ was added to the co-cultures. NK cell IL-10 secretion was significantly reduced in the cultures treated with IFNγ (Fig 3J). To confirm these effects were due to IFNγ signaling in the NK cells, we established co-cultures using B6.*il10* ^-/-^ BMDC and NK cells purified from spleens of B6.*ifngr1* ^-/-^ mice. The IFNGR-deficient NK cells produced 4–5 fold more IL-10 than wt B6 NK cells (Fig 3K). Secreted IL-10 was also detected earlier in the cultures with IFNGR-deficient NK cells (Fig 3L). These results suggest that early IFNγ production may contribute to the observed delay in IL-10 production by the responding NK cells.

### NK cell IL-10 production increases susceptibility to systemic Lm infection

**Fig 4:**
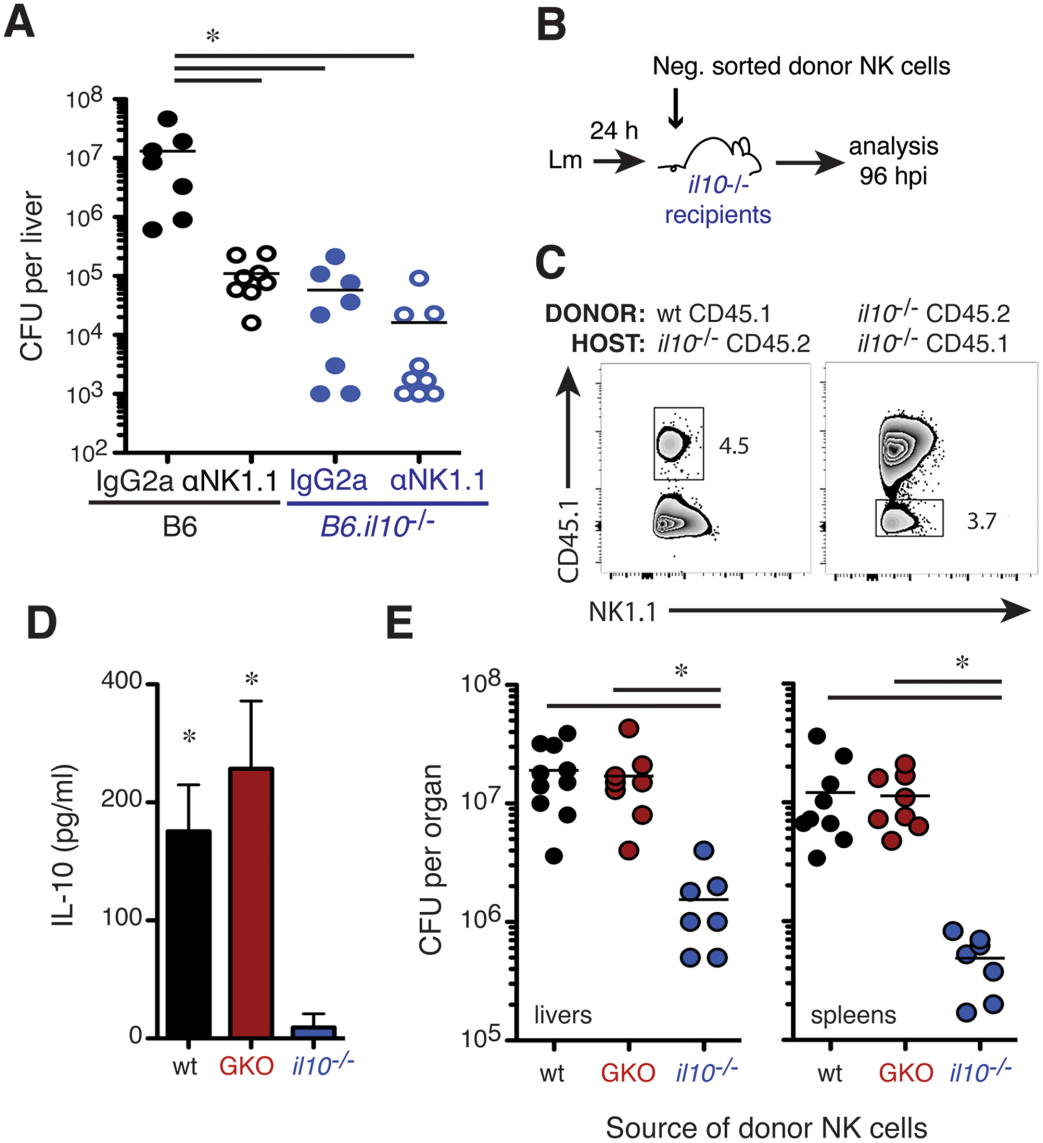
NK cell IL-10 production drives increased Lm burdens. (**A**) Liver CFU from individual B6 and congenic IL-10 deficient B6 (*B6*.*il10* ^*-/-*^) mice at 72 hpi. IgG2a or αNK1.1 Abs were given 24 h before infection. Pooled from two experiments representing n = 7–9 mice/group. (**B**) Design of cell transfer experiments. NK cells were purified from naïve wt CD45.1^+^ (B6.*ptprc* ^*a*^), IL-10-deficient CD45.2^+^ (B6.*il-10* ^*-/-*^), or IFNγ-deficient (GKO) CD45.2^+^ mice for transfer into infected B6.*il-10* ^*-/-*^ recipients. (**C**) Detection of CD45.1^+^ donor NK cells in B6.*il10* ^*-/-*^ recipients at 96 hpi. Live gated CD3^-^NK1.1^+^ splenic NK cells are shown. (**D**) IL-10 from splenic lysates of infected B6.*il-10* ^*-/-*^ recipients 72 h after NK cell transfers. Shown are mean ± SEM for one of three experiments using n = 3–4 mice/group. (**E**) Lm burdens from indicated tissues of B6.*il-10* ^*-/-*^ recipients 96 hpi. *P< 0.05 as determined by (A) ANOVA or (D, E) t-test.

To further investigate the relationship between pro-bacterial effects of NK cells and their production of IL-10, NK cell depletion was performed in IL-10-deficient (B6.*il10* ^-/-^) mice. Analysis of the infected B6.*il10* ^-/-^ animals revealed that liver bacterial burdens were comparable to those seen in B6 mice depleted of NK cells prior to infection (Fig 4A). NK cell depletion failed to further reduce Lm burdens in the B6.*il10* ^-/-^ mice. Thus, NK cell depletion and IL-10 deficiency had similar and non-additive effects on susceptibility to Lm, suggesting production of IL-10 by NK cells might be responsible for the increased host susceptibility. To further test this, we performed adoptive transfer experiments. CD45.2^+^ or CD45.1^+^ B6.*il10* ^-/-^ recipients were infected with Lm then respectively transferred with NK cells from the spleens of naïve wt CD45.1 (B6.*ptprc* ^*a*^) or IL-10 deficient CD45.2 (B6.*il10* ^-/-^) mice (Fig 4B). At 96 hpi (72 h after transfer) small populations of donor CD45.1^+^ wt and CD45.2^+^ IL-10 deficient NK cells could be detected in spleens of the B6.*il10* ^-/-^ CD45.2^+^ and CD45.1^+^ recipients, respectively (Fig 4C), demonstrating persistence of the transferred cells. The detection of IL-10 protein in lysates of splenocytes from the B6.*il10* ^-/-^ recipients of wt, but not IL-10-deficient, NK cells indicated the transferred cells were activated to produce IL-10 (Fig 4D). This IL-10 production in the presence of wt NK cells was also associated with 10–100 fold increases in Lm burdens in livers and spleens of the B6.*il10* ^-/-^ mice (Fig 4E).

To establish whether IFNγ production by the NK cells might mediate pro- or anti-bacterial effects in the recipient mice, groups of B6.*il10* ^-/-^ mice received NK cells from the spleens of IFNγ deficient (GKO, B6.*ifng* ^-/-^) mice. The GKO NK cells produced IL-10, as measured in spleen lysates (Fig 4D), and GKO NK cells sufficed to increase Lm burdens (Fig 4E). There was no significant difference in burdens of mice receiving wt or GKO NK cells and both NK cell types increased burdens in the *il10* ^-/-^ mice to a level near that seen in wt mice infected with Lm (compare Fig 4A and 4E). Experiments using donor NK cells labeled with CFSE further confirmed that the take of GKO, WT, and *il10* ^-/-^ NK cells was similar in the *il10* ^-/-^ recipients (S3A Fig). Thus, the ability of NK cells to produce IL-10 is a crucial factor governing Lm survival and replication during systemic infection and these pro-bacterial effects are independent from NK cell production of IFNγ.

### NK cells selectively regulate inflammatory myeloid cell responses

**Fig 5:**
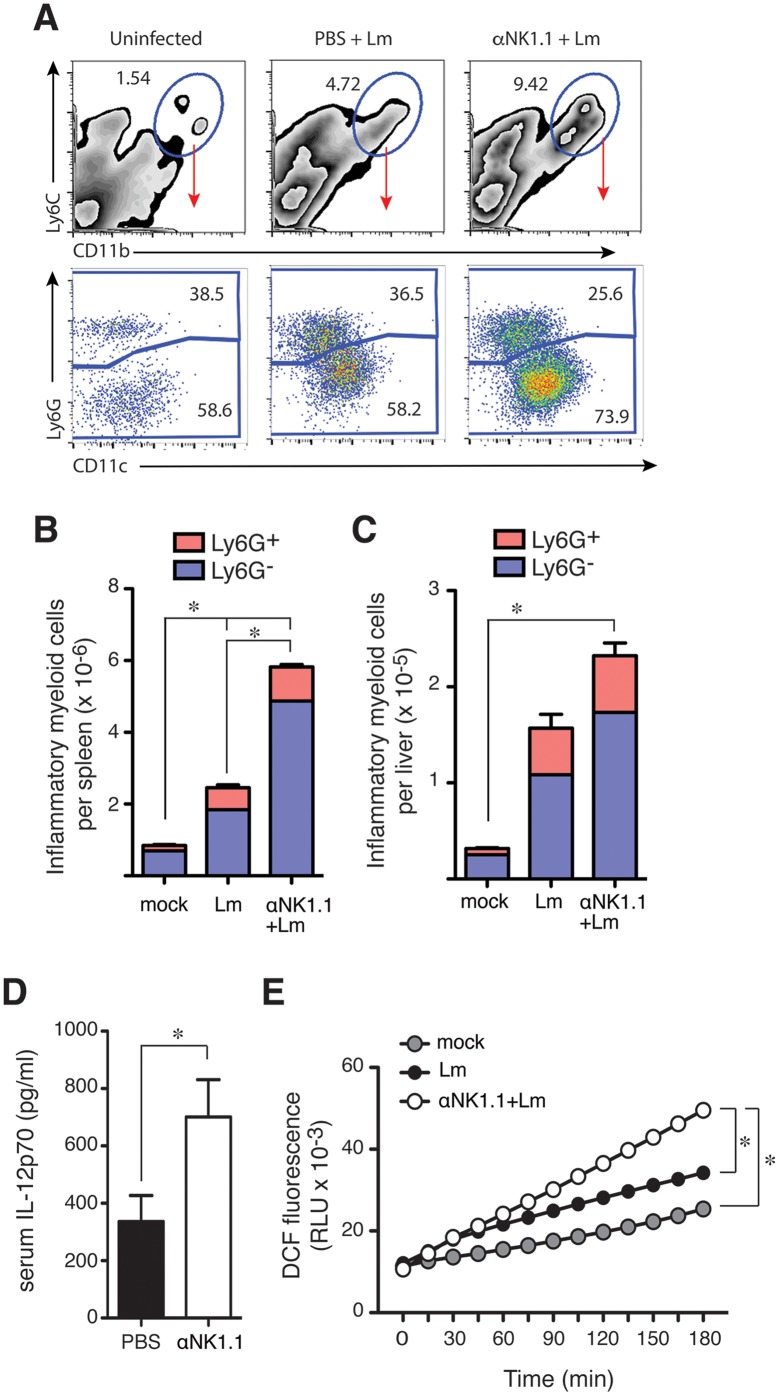
NK cells suppress innate responses and their production of IL-10 increases susceptibility to Lm. (**A**) Splenocytes were harvested from uninfected or 72 hpi B6 mice and evaluated for the indicated myeloid cell markers. Top row depicts total live-gated population bottom row depicts the indicated gated population. Data are from individual mice and representative of more than n = 20 mice from over 5 experiments. (**B**) Splenocytes were harvested and analyzed as above. Shown are the mean ± SEM number of each indicated cell population per spleen as calculated from data gated as in (A). Data are from one of more than 5 experiments and represent n = 5 individual mice per group. (**C**) Leukocytes were isolated from livers of the indicated mice and analyzed as above. Shown are the mean ± SEM number of each indicated cell population per liver for one of 3 experiments and represent n = 5 individual mice per group. (**D**) Serum IL-12p70 for control IgG2a and αNK1.1-treated mice at 96 hpi. Shown are mean ± SEM from four pooled experiments representing n = 12–16 mice/group. (**E**) DCF fluorescence was measured at the indicated times after plating of labeled splenocytes harvested from uninfected (mock) or Lm-infected control IgG2a and αNK1.1 treated mice at 72 hpi. Shown are mean fluorescence values for triplicate wells from one of three experiments. *P<0.05 as measured by (B, C) ANOVA, (D) t-test, or (E) 2 way ANOVA.

IL-10 is known to suppress M1-type myeloid cell activation as well as the production of IFNγ by activated T cells [15]. Activated myeloid and T cells are both important for mediating resistance to Lm infection [2,3]. Thus, we asked whether NK cell IL-10 production might suppress myeloid or T cell responses during Lm infection. A Ly6C^+^CD11b^+^ inflammatory myeloid cell population was observed to accumulate by 3–4 dpi with Lm infection in both control and NK cell-depleted mice (Fig 5A, top). In both cases, the accumulating cells were a mixture of Ly6G^+^ neutrophils and Ly6G^-^CD11c^l^° inflammatory monocytes (Fig 5A, bottom). However, we observed significantly more of these inflammatory cells in spleens (Fig 5B) and livers (Fig 5C) of the mice lacking NK cells. Increased numbers of Ly6C^+^CD11b^+^ cells were also seen in spleens of Lm-infected *il10* ^-/-^ mice and these numbers were reduced when mice received NK cells capable of producing IL-10 during the experiments shown in Fig 4 (S3B Fig). These results suggest NK cell IL-10 suppresses the accumulation of these inflammatory myeloid cell populations at sites of bacterial infection. Both Ly6G^-^ inflammatory monocytes and Ly6G^+^ granulocytes can ingest and kill bacteria to mediate protection against Lm [30,31]. Thus, blunting the accumulation of these inflammatory cell populations might itself promote bacterial infection. NK cell IL-10 also appeared to suppress the activation of these recruited myeloid cells, as we observed increased serum concentrations of IL-12p70 (a product of activated myeloid cells) in mice depleted of NK cells (Fig 5D). Further, *in vivo* depletion of NK cells before Lm infection led to an increased production of reactive oxygen species (ROS) by adherent splenocytes cultured from spleens of infected mice as measured by increased fluorescence of the ROS-sensitive dye, DCF (Fig 5E). These data together suggest regulatory NK cell activity suppresses accumulation and activation of inflammatory myeloid cells and thus their ability to ingest and kill Lm or infected cells.

In contrast to the effects on myeloid cell responses, depleting NK cells did not notably affect the activation of T cells specific for Lm-expressed antigens when measured at 7 dpi or following secondary Lm challenge (S4 Fig). Here, mice were treated with control and αNK1.1 antibodies before immunization with an ovalbumin expressing Lm strain (Lm-OVA). NK cell depletion did not change the number of Lm- or OVA- peptide responsive CD4^+^ or CD8^+^ T cells that stained positive for intracellular IFNγ^+^ 7 d later (S4A–S4C Fig). We also failed to observe altered expansion of T cells in response to secondary Lm challenge and the NK cell-depleted mice developed protective immunity (S4D and S4E Fig). These results suggest that the increased bacterial burdens associated with NK cell regulatory activity are primarily due to suppression of inflammatory myeloid cell, but not T cell, mediated immune responses.

## Discussion

Lm infection elicits a potent NK cell response, but whether and how NK cells might impact host resistance to Lm and other bacteria has remained poorly understood. One notion often stated in the literature is that NK cells protect against bacterial infections through their production of IFNγ. Certainly, IFNγ is crucial for resistance to Lm [32]. However, as shown here, NK cell IFNγ production peaks and wanes rapidly. T cells were also a significant early source of IFNγ production in our studies, and memory-phenotype T cells are known to be capable of antigen-independent IFNγ production that can mediate protection against Lm [7]. Memory-phenotype T cells transferred into IFNγ^-/-^ mice conferred significantly more protection than transferred NK cells, though the latter conferred a low degree of protection in the absence of any other source of IFNγ [33]. Thus, IFNγ production by T cells appears to obviate IFNγ production by NK cells. Consistent with this interpretation, depleting NK cells in wildtype mice did not increase Lm burdens. Instead, we confirmed several older reports showing that depletion of NK1.1^+^ cells reduces severity of systemic infections by this and other bacteria [14,34,35]. The older studies did not discriminate the effects of NK versus NKT cells, but we showed here that the absence of NKT cells had no effect on Lm burdens during systemic infection. These results support the conclusion that NK cells are not a crucial source of early IFNγ production and that NK cells instead mediate pro-bacterial effects. Our data further demonstrate the mechanism responsible for these pro-bacterial effects. We found that NK cells responding to Lm produce IL-10 and this production is necessary and sufficient to increase Lm burdens. Further, the p60 virulence protein of Lm drives NK cell activation and IL-10 production. NK cells consequently suppressed innate myeloid cell responses. These findings together suggest Lm promotes NK cell activation to exploit their regulatory effects on antibacterial myeloid cell responses.

Despite the evidence that NK cell activation has deleterious effects during systemic Lm infection we are aware of a few seemingly contradictory reports. In studies where Lm was introduced into the footpad of mice, NK cell depletion was shown to modestly increase bacterial burdens in the draining lymph nodes [36,37]. Lm does not normally infect the host through the skin and little is known about the sequence of immune responses in this model. Perhaps footpad-inoculated Lm is unable to exploit NK cell IL-10 production, for example due to differences in the pattern or kinetics of inflammatory cell recruitment. Another example where NK cell deficiency was reported to increase susceptibility was in mice doubly deficient for the common gamma chain (γ_c_) and Rag2 [38]. The γ_c_ is important for cellular responses to several cytokines, including IL-2, 4, 7, 9, 15 and 21. IL-15 signaling is particularly important for development and survival of NK and memory CD8^+^ T cells [39,40]. The increased susceptibility in the γ_c_^-/-^ x *rag2* ^-/-^ mice might thus be interpreted to indicate a protective role for NK cells. However, mice singly deficient for γ_c_ or Rag2 did not demonstrate increased susceptibility to Lm infection [38]. Thus, the reported susceptibility of mice doubly deficient for Rag2 and γ_c_ is not simply a result of NK cell deficiency. It was suggested that NK cells may be a key source of protective IFNγ in the absence of T cells [38]. It is also notable that recent studies indicate that Rag protein expression in NK cells modulates their survival and functional activity [41]. Regardless, it has since been shown that IL-15^-/-^ mice exhibit increased resistance to systemic Lm infection [42].

It has been previously postulated that NK cells might exacerbate Lm infection through overproducing IFNγ [14]. Three lines of evidence from our studies argue against this model: (1) We showed that mice lacking IFNGR1 expression were still protected by depletion of NK1.1^+^ cells despite their increased susceptibility overall. (2) Depletion of NK cells was protective when initiated subsequent to peak NK cell IFNγ production. (3) Transfer of GKO NK cells were as effective as wt NK cells at increasing Lm burdens in IL-10 deficient recipients. However, Jablonska and colleagues recently argued that NK cell secretion of IFNγ contributes to pro-bacterial effects based on the finding that mice treated with a low dose of anti-IFNγ antibody had heightened resistance [43]. These apparently discrepant results could be explained by antibody stabilization of IFNγ to enhance its signaling, as has been shown to occur when cytokines such as IL-2 and IL-15 are complexed with antibodies or soluble receptor subunits [44,45]. We thus interpret the available data as evidence that NK cells exert pro-bacterial effects independent from their IFNγ production.

We instead found that the key mechanism by which NK cells increase susceptibility to Lm is through production of IL-10. How does NK cell-derived IL-10 suppress resistance to bacterial infection? It is well established that mice entirely deficient for IL-10 are highly resistant to Lm infection. This resistance is associated with increased innate and adaptive immune responses [17]. Consequently, we evaluated the effects of NK cell depletion on both T and myeloid cell responses to Lm infection. We failed to observe any effect of NK cells on the T cell response to Lm infection, suggesting that NK cell IL-10 is not responsible for the previously observed suppression of T cell responses. Consistent with this result, CD4^+^ T cells were recently shown to be a crucial source of IL-10 that regulates memory CD8^+^ T cell responses during LCMV infection [46]. In contrast, our findings here indicated that NK cell IL-10 suppresses both accumulation and activation of myeloid cells. Coincident with the timing of NK cell IL-10 production in control Lm infected mice (3–4 dpi), we found that depleting NK cells increased inflammatory monocyte and neutrophil accumulation in spleens and livers, increased serum IL-12p70, and increased ROS production in cultures of adherent splenocytes. Activated myeloid cells are the primary source of IL-12, and its production is known to be suppressed by IL-10 [47,48]. ROS production is also a correlate of M1-type activation, is suppressed by IL-10, and correlates with macrophage and neutrophil bactericidal activity [49,50]. Further, IL-10 blockade is known to increase macrophage bactericidal activity against Lm [49,51,52]. Production of IL-12 or ROS may not themselves mediate reduced Lm burdens in NK cell-depleted mice, but certainly indicate enhanced myeloid cell activation. Accumulation and activation of myeloid cells is crucial for immune resistance to infections by Lm and many other intracellular pathogens [1]. Hence, the impairment of these processes likely accounts for the ability of NK cell IL-10 to increase susceptibility during Lm, and possibly other, bacterial infections. Presumably, this dampening of inflammatory responses benefits the host in other settings, such as in the context of inflammatory diseases.

Lm infection is not unique in its ability to stimulate IL-10 secretion by NK cells. NK cell IL-10 production was previously observed at late stages of chronic infection by *Leishmania donovanni* [19], and during infections by *Toxoplasma gondii* [18], and murine cytomegalovirus (MCMV) [53]. During *T*. *gondii* infection, NK cell activation is regulated by cytokine (IL-12) production and this IL-12 is driven by ligation of TLR11 and 12 by the parasite profilin protein [54]. IL-12 production during *T*. *gondii* [18] induces NK cell expression of the aryl hydrocarbon receptor (Ahr) transcription factor to drive *il10* transcription [55]. IL-12 also drives *il10* transcription in NK cells during chronic infection by the parasite *L*. *donovanii* and IL-10 producing NK cells were shown to increase parasite numbers in this model [19]. However, it is not yet clear whether a specific *L*. *donovanii* protein drives the NK cell response to this infection. NK cell IL-10 production during MCMV infection is largely seen in the Ly49H^+^ NK cell subset [25]. Ly49H is an activating receptor that responds to the virus-encoded M157 protein [56,57]. Work here showed that the Lm p60 protein was important for promoting regulatory NK cell activity. Our prior work found that Lm expression of the p60 protein increases both bacterial replication in host tissues and NK cell production of IFNγ early after systemic infection [5]. Recombinant p60 protein was subsequently shown to stimulate production of IL-18 by primed BMDC to stimulate cell contact-dependent NK cell production of IFNγ [11,58]. In the present paper Lm expression of p60 increased NK cell secretion of IL-10 *in vivo* and in BMDC/NK cell co-cultures. Stimulation of primed murine BMDCs or human PBMC-derived DCs with a recombinant fragment of p60 protein (L1S) likewise sufficed to trigger NK cell secretion of IL-10. These data suggest Lm uses p60 to actively promote NK cell IL-10 secretion. Together with the prior work in other pathogen models, these data with p60 further suggest that the presence of NK cell stimulating proteins might be a marker for pathogens that have evolved to exploit NK cell regulatory activity.

The kinetics of NK cell IL-10 secretion was found here to occur only after reductions in NK cell IFNγ secretion, both during systemic infection and in cell co-cultures infected with Lm or stimulated with recombinant L1S protein. Similar delays were observed in other models where NK cell IL-10 production occurs [18,19,53]. For example, during *Leishmania donovani* infection NK cell IFNγ production is stimulated in an IL-12-dependent manner from 24 hr infected mice, followed by NK cell IL-10 production from 21 day infected mice [19]. Similarly, during MCMV infection IL-2 and IL-12 drove NK cell IFNγ as well as subsequent IL-10 production in culture ex vivo [53]. These studies suggest that the switch from IFNγ to IL-10 production is a consequence of NK cell activation in multiple infections. However, we do not believe this switch is a “hard-wired” response to activation given previous reports showing certain cytokine stimulation protocols which induce IFNγ production by human NK cells fail to also trigger IL-10 secretion [59,60]. Recent work from Biron and colleagues suggested that during MCMV infection this delay reflects a requirement for NK cell proliferation to open the *il10* locus to transcriptional machinery [53]. Proliferation might similarly contribute to IL-10 production by NK cells during Lm or other infections, though this remains to be determined. Defining the mechanistic basis for sequential production of IFNγ and IL-10 was not the purpose of our studies, but we did observe that IFNγ acts to suppress NK cell IL-10 secretion. Further, we found that NK cells deficient in IFNγ signaling secreted increased quantities of IL-10. These results suggest early NK cell IFNγ production may be important for suppressing or delaying NK cell IL-10 secretion. In T cells an initial pro-inflammatory response is necessary for switching from IFNγ to IL-10 production [61–63]. However, we found that IFNγ-deficient NK cells remained capable of producing IL-10 and were as effective as wt NK cells at increasing susceptibility in IL-10^-/-^ mice. Thus, cell-intrinsic IFNγ production does not appear to be an essential stimulus for NK cell IL-10 secretion. What additional factor(s) might contribute to driving NK cell transitioning from IFNγ to IL-10 production during Listeria and other infections remains to be determined.

The impact of NK cell regulatory activity on human health and disease is not yet known. However, human NK cells were previously shown to produce IL-10 [59], and we showed that Lm p60 could drive IL-10 secretion by human NK cells. Thus, NK cell IL-10 production may well impact human susceptibility to infections and other diseases, including Lm infection. Severe Lm infections primarily occur in elderly and pregnant individuals. Ageing is associated with an increase of circulating NK cells in humans [64], and NK cells are a major cell population in the placenta of pregnant humans and animals [65]. Perhaps then, the increased prevalence of NK cells and their IL-10 production is an important factor governing the susceptibility of these populations. Pregnant individuals also have increased susceptibility to infections by *T*. *gondii*, *Cytomegalovirus*, and *Leishmania* [66]. Depletion of NK cells or more selective approaches to suppress their acquisition of regulatory activity could thus prove useful in some clinical settings. Improved understanding of how Lm p60 and other pathogen factors induce pro-inflammatory and regulatory NK cell responses will be an important step in defining the potential benefits, risks, and feasibility of manipulating NK cells in the context of infectious, autoimmune, and cancerous diseases.

## Methods

### Animals

Female mice were used at 8–12 weeks of age. C57BL/6J, *B6*.*il10* ^-/-^, *B6*.*ptprc* ^*a*^, B6.*ifngr1* ^-/-^, B6.*ifnar1* ^-/-^, B6.*ifng* ^-/-^ (GKO) and B6.IL-10-gfp (tiger) mice were purchased from Jackson Labs. *B6*.*cd1d* ^-/-^ mice were from Dr. Laurent Gapin (Univ. Colorado). Mice were maintained in the National Jewish Health Biological Resource Center and University of Colorado Office of Laboratory Animal Resources.

### NK cell depletion

Mice were treated *i*.*p*. with PBS or PBS containing 100 μg of purified Ab or 100 μl of rabbit antisera to the ganglioside asialoGM1 (α-GM1, Wako USA). Endotoxin free IgG2a control (C1.18) and αNK1.1 (PK136) Abs were purchased (Bio X Cell). Anti-Ly49C/I (clone 5E6) and anti-NKG2A/C/E (20D5) Abs were purified from hybridoma supernatants using protein A affinity chromatography. Unless otherwise stated, Abs were given in a single dose at 24 h before infection. Depletions were confirmed by flow cytometry.

### Infections

*L*. *monocytogenes* (Lm; strain 10403s), congenic p60-deficient [28], and OVA-expressing [67] Lm (OVA-Lm) were thawed from frozen stocks and diluted for growth to log phase in brain-heart infusion or tryptic soy broth (MP Biomedicals), then diluted in PBS and given to mice *i*.*v*. in the lateral tail vein. Unless stated otherwise, mice received a single sublethal dose of 10^4^ CFU. Lm was given at 4000 CFU for infection in B6.*ifngr1* ^-/-^ mice, and a lethal dose of 5 x 10^4^ CFU for analysis of survival ± NK cell depletion. OVA-Lm was given at 5000 CFU for immunizations. Challenges used a lethal dose of Lm-OVA (10^5^ CFU). For CFU counts, organs were harvested into 0.02% Nonidet P-40, homogenized for 1 min with a tissue homogenizer (IKA Works, Inc.) and serial dilutions were plated on BHI or TSB agar plates.

### Cell isolation and stimulation

Spleens were harvested into media containing penicillin/streptomycin at 100 U/ml then transferred to a solution of 1 mg/ml type 4 collagenase (Worthington) in HBSS plus cations (Invitrogen). After a 30 min incubation at 37°C, spleens were disrupted by passage through a 70 μm cell strainer and the resulting single cell suspensions treated with RBC lysis Buffer (0.15 M NH_4_Cl, 10mM KHCO_3_, 0.1 mM Na_2_EDTA, pH 7.4) for 2 min. Prior to intracellular staining, splenocytes were incubated 3–4 h in RP10 media (RPMI 1640, 10% FBS, 1% L-glutamine, 1% Sodium Pyruvate, 1% Penicillin, 1% Streptomycin and 0.1% β-ME) containing Brefeldin A (GolgiPlug; BD Biosciences). No additional *ex vivo* stimulation was included for NK cell analyses. For T cells, 1 μM concentrations of synthetic OVA_257–264_ (SIINFEKL) or LLO_190–201_ (NEKYAQAYPNVS) peptides were included during the incubation. Blood cells were harvested into HBSS plus cations and heparin (Sigma). Cells were treated twice with RBC lysis Buffer for 1 min. Liver cells were harvested and treated with collagenase in the same manner as spleen cells. Following passage through a 70 μm cell strainer, cells were re-suspended in 40% Percoll in HBSS minus cations. The 40% Percoll was underlayed with 60% Percoll, and cells were collected from gradient following centrifugation. RPMI with 5% FBS was added to cells to pellet, and cells were treated with RBC lysis Buffer for 1 min.

### Flow cytometry

Anti-CD16/32 (2.4G2 hybridoma supernatant) was added to block Fc receptors prior to staining, which used FACS buffer (1% BSA, 0.01% NaN3, PBS) containing fluorophore-labeled antibodies purchased from eBioScience or BioLegend. Staining antibodies included anti- CD3 (clone 145 2C11), CD4 (clone RM-4-5), CD8 (clone 53–6), CD11b (M1/70), CD11c (N418), CD27 (clone LG.7F9), CD45.1 (clone A20), IFNγ (clone XMG1.2), Ly6C (HK1.4), Ly6G (1A8), NK1.1 (clone PK136), and NKp46 (clone 29A1.4). After surface staining, cells were fixed in 2–4% paraformaldehyde for direct analysis with or without saponin treatment for intracellular staining. To amplify IL-10-gfp signal [53], fixed and permeabilized cells were stained with a rabbit monoclonal anti-GFP followed by goat anti-rabbit IgG Alexa Fluor 488 (both from Life Technologies). At least 100,000 data events per sample were collected using an LSRII (BD Biosciences). FlowJo software (Treestar) was used for analysis of flow data.

### Co-culture system

Bone marrow-derived DC (BMDC) were cultured from B6.*il10* ^-/-^ mice and infected with Lm or stimulated with recombinant L1S protein purified as previously described [11,29]. Briefly, bone marrow was cultured 6d in GM-CSF and 3 x 10^5^ BMDC (>90% CD11c^+^) per well were cultured overnight in 24 well plates. For infections, log phase wt or Δp60 Lm were added at a multiplicity of one bacterium per BMDC. One h later, cells were washed and gentamycin was added at 10 μg/mL. For L1S stimulation, BMDC were activated 1 h by treatment with 20 μg/ml poly I:C (Invivogen, San Diego, CA) or 10 ng/ml LPS (L8274 Sigma-Aldrich, St. Louis, MO) and purified L1S protein was added to the BMDC at 30 μg/ml. For IFNγ treatment, 50 ng/mL IFNγ (Life Technologies) was added at 1 hr post L1S stimulation. Purified splenic NK cells were negatively sorted using the EasySep Mouse NK cell Enrichment Kit (19755 Stemcell Technologies) and added to cultures 2 h after Lm or L1S treatments at a ratio of .1:1 (NK cells:BMDC). Purified NK cell populations were >80% NK1.1^+^CD3^-^. To culture human DCs, adherent PBMCs were obtained from unrelated donors and grown in RPMI 1640 (Invitrogen) supplemented with 10% human AB serum (Innovative Research, Novi, MI), 0.01M HEPES, 0.02mg/ml gentamicin, 200 IU/ml IL-4 (eBioscience), and 100 IU/ml GM-CSF (R&D Systems, Minneapolis, MN). After 6 days, DC were plated at 10^5^ cells/well in 96 well plates, primed, and stimulated with L1S and polyI:C as above. Purified human NK cells were negatively sorted from PBMCs using the EasySep Human NK cell Enrichment Kit (19015 Stemcell Technologies). Supernatants were harvested for analysis of IFNγ and IL-10 at 24 or 72 h cytokines using commercial ELISAs (BD Biosciences).

### Cytokine analysis

Blood was collected by cardiac puncture. Serum from clotted blood was collected and frozen prior to analysis using commercial ELISAs for IFNγ, IL-12p70, or IL-10 (BD Biosciences). Equal numbers of splenocytes were homogenized in 1 mL of 0.02% Nonidet P-40 with protease inhibitor cocktail (ThermoFisher) using a dounce for measurement of IL-10 in homogenates.

### Adoptive transfers

NK cells were purified from spleens of naïve B6.*ptrpc* ^*a*^, B6.*il10* ^-/-^, and B6.*ifng* ^-/-^ (GKO) mice (>80% purity) using the EasySep Negative Selection Mouse NK Cell Enrichment Kit (Stemcell Technologies). Each recipient received 1.5–2 x 10^6^ live NK cells *i*.*v*. at 24 hpi.

### ROS analysis

Single cell splenocyte suspensions were prepared as above and 10^6^ cells were added per well to a 96 well round bottom suspension plate (Greiner) followed by centrifugation at 500 x g for 5 min. Pelleted splenocytes were resuspended in DPBS (Gibco) containing 10μM 2',7'-dichlorodihydrofluorescein diacetate (DCF; ThermoFisher) and 1% DMSO. After a 30 min incubation at 37°C, spenocytes were washed twice in DPBS and resuspended at 100 μl/well in phenol red-free DMEM (Gibco) then transferred to 96 well white bottom Nunc F96 plates (ThermoFisher). Plates were incubated in a 37°C Biotek Synergy HT plate reader and fluorescence read at 15 min intervals using a 528/20 emission filter and Gen5 software.

### Statistics

Graphing and statistical analyses used Prism (GraphPad Software). Statistical tests included t-tests, analysis of variance (ANOVA), and linear regression with Pearson correlation. P<0.05 was considered significant.

### Study approval

The Animal Care and Use Committees for National Jewish Health (Protocol# AS2682-08-16) and the University of Colorado School of Medicine (Protocol# 105614(05)1E) approved all studies. These protocols adhere to standards of the United States Public Health Service and Department of Agriculture.

## Supporting Information

S1 Fig Effectiveness of NK cell depletion using αNK1.1, impact of αLy49C/I, and sources of intracellular IFNγ. (**A**) Eight week old female C57BL/6 mice were given a single *i*.*p*. injection of PBS containing 100 mg of purified IgG2a control (C1.18) or αNK1.1. Twenty-four h later (0 hpi), spleens were harvested or mice were infected i.v. with 10^4^ log-phase Lm. Depletion of splenic NK1.1^+^NKp46^+^ NK cells was evaluated at 0 and 96 hpi. Shown are representative plots of CD3^-^ gated splenocytes. (**B**) Groups of B6 mice treated with purified IgG2a control or αNK1.1 and infected with 5000 CFU Lm. Bacterial burdens were determined at 96 hpi. Symbols represent individual mice from one of two experiments using n = 5–7 mice/group. (**C**) Groups of B6 mice were treated with purified IgG2a isotype control, αNK1.1, or a depleting αLy49C/I monoclonal Ab (clone 5E6) and infected with 10^4^ Lm. Bacterial burdens were determined at 96 hpi. Symbols represent individual mice from one of two experiments using n = 4–5 mice/group. *P<0.05 by ANOVA. (**D**) Representative flow cytometry data plots showing intracellular IFNγ^+^ (iIFNγ^+^) cell populations present in the spleen of a B6 mouse at 24 hpi with Lm. Panels on the left show live gated cells. The right panel depicts staining of the gated iIFNγ^+^ population indicated with the red arrow. (**E**) Total cell numbers of IFNγ^+^NK1.1^+^CD3^-^ cells present in the spleens of mock-infected or Lm-infected mice. Symbols represent individual mice from one of two experiments using n = 4–5 mice/group. (**F**) Total cell numbers of IFNγ^+^CD3^+^ cells present in the spleens of mock-infected or Lm-infected mice. Symbols represent individual mice from one of two experiments using n = 4–5 mice/group.
(TIF)

S2 Fig Flow cytometry plots for detection of NK cell co-expression of IFNγ and il10-gfp reporter. Representative flow cytometry data plots show iIFNγ and il10-gfp staining on CD3^-^NK1.1^+^ gated cells isolated from the spleen, liver, and blood of tiger IL-10 GFP-reporter mice at 24 or 72 hpi with 10^4^ Lm.
(TIF)

S3 Fig Detection of CFSE-labeled cells and inflammatory myeloid cells in mice that received purified NK cells. **(A)** CFSE and NK1.1 staining on CD3^-^NK1.1^+^ gated cells isolated from the spleens of IL-10^-/-^ recipient mice at 72 hr post transfer of CFSE-labeled purified NK cells and 96 hpi with 10^4^ Lm. **(B)** Total numbers of Ly6C^+^CD11b^+^ inflammatory myeloid cells per spleen at 96 hpi in IL-10^-/-^ mice that received NK cells purified from wt, *il10* ^*-/-*^, or GKO mice. *P < .05 by ANOVA.
(TIF)

S4 Fig NK cell depletion does not impair T cell responses or development of long term protective immunity. (**A-C**) Control IgG2a or αNK1.1 Abs were given 24 h before priming of mice with live Lm-OVA (5000 CFU). At 7 dpi splenocytes were harvested and stimulated with synthetic peptides corresponding to Lm-derived epitopes from ovalbumin (OVAp) or LLO (LLOp). (A) Intracellular IFNγ in gated CD3^+^8^+^ splenocytes. (B) Intracellular IFNγ in gated CD3^+^4^+^ splenocytes. (C) Numbers of responding CD4^+^ and CD8^+^ cells per spleen calculated from the respective gated iIFNγ^+^ populations. Symbols represent values from individual mice and horizontal lines indicate means. Shown are data from one of two experiments using n = 4 mice/group. (**D-E**) Mice immunized as above were re-challenged using a high dose (10^5^ CFU) of Lm-OVA 28 d later. (D) Intracellular IFNγ in gated CD3^+^4^+^ and CD3^+^8^+^ T cells from spleens of the re-challenged mice. Staining was done at 96 h after challenge. Plots are representative of 4–5 mice/group. (E) Liver Lm burdens from mice in (D). Age-matched control mice received challenge without prior immunization. *P < .05 by ANOVA.
(TIF)
