# Supplementary figures and images for: Bacterial Manipulation of NK Cell Regulatory Activity Increases Susceptibility to Listeria monocytogenes Infection
Source: PLoS Pathog. 2016 Jun 13;12(6):e1005708. doi: 10.1371/journal.ppat.1005708 (PMC4905663; doi:10.1371/journal.ppat.1005708)

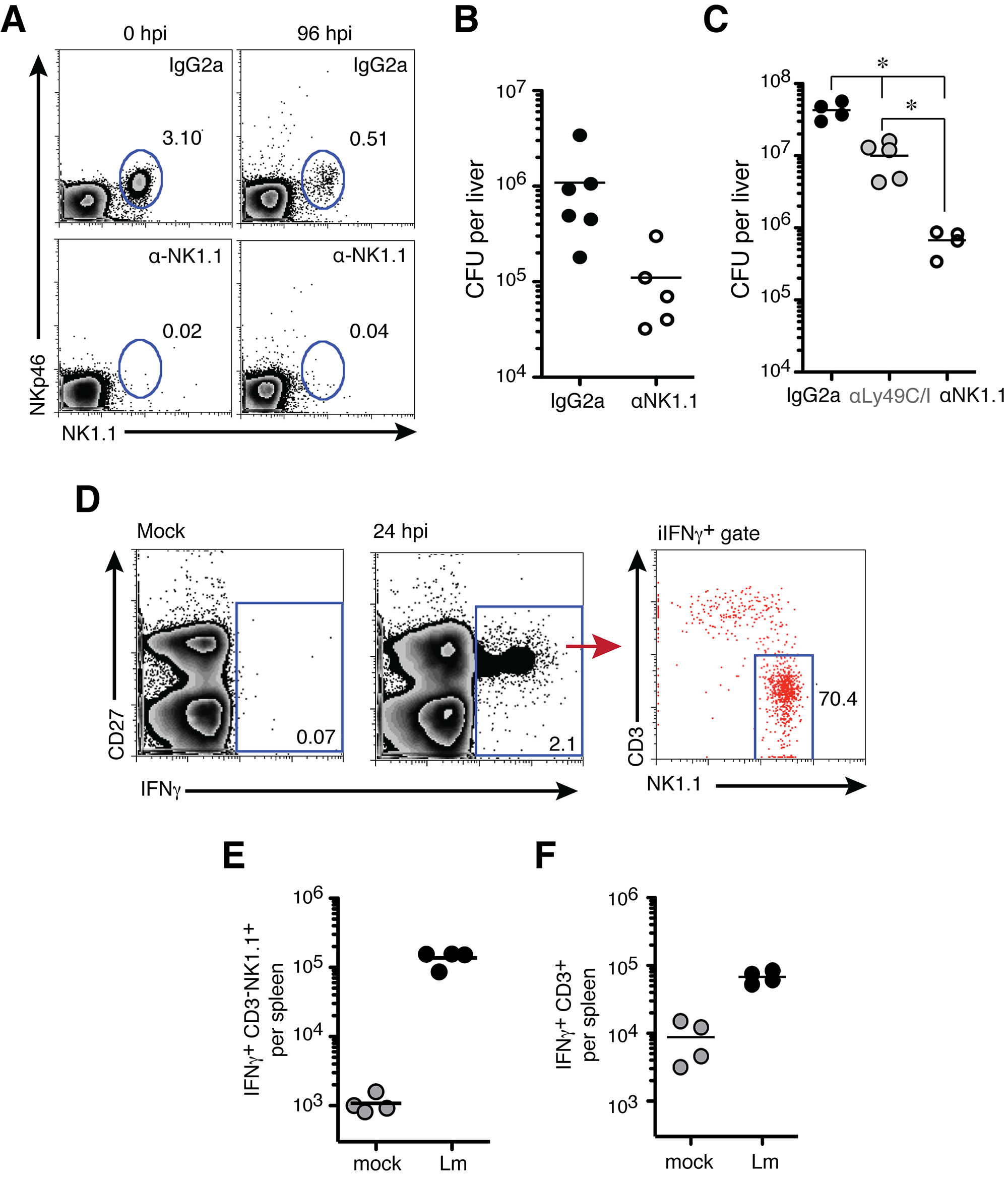

Supplement: S1 Fig — (A) Eight week old female C57BL/6 mice were given a single i.p. injection of PBS containing 100 mg of purified IgG2a control (C1.18) or αNK1.1. Twenty-four h later (0 hpi), spleens were harvested or mice were infected i.v. with 104 log-phase Lm. Depletion of splenic NK1.1+NKp46+ NK cells was evaluated at 0 and 96 hpi. Shown are representative plots of CD3- gated splenocytes. (B) Groups of B6 mice treated with purified IgG2a control or αNK1.1 and infected with 5000 CFU Lm. Bacterial burdens were determined at 96 hpi. Symbols represent individual mice from one of two experiments using n = 5–7 mice/group. (C) Groups of B6 mice were treated with purified IgG2a isotype control, αNK1.1, or a depleting αLy49C/I monoclonal Ab (clone 5E6) and infected with 104 Lm. Bacterial burdens were determined at 96 hpi. Symbols represent individual mice from one of two experiments using n = 4–5 mice/group. *P<0.05 by ANOVA. (D) Representative flow cytometry data plots showing intracellular IFNγ+ (iIFNγ+) cell populations present in the spleen of a B6 mouse at 24 hpi with Lm. Panels on the left show live gated cells. The right panel depicts staining of the gated iIFNγ+ population indicated with the red arrow. (E) Total cell numbers of IFNγ+NK1.1+CD3- cells present in the spleens of mock-infected or Lm-infected mice. Symbols represent individual mice from one of two experiments using n = 4–5 mice/group. (F) Total cell numbers of IFNγ+CD3+ cells present in the spleens of mock-infected or Lm-infected mice. Symbols represent individual mice from one of two experiments using n = 4–5 mice/group. (TIF) [file ppat.1005708.s001.tif]

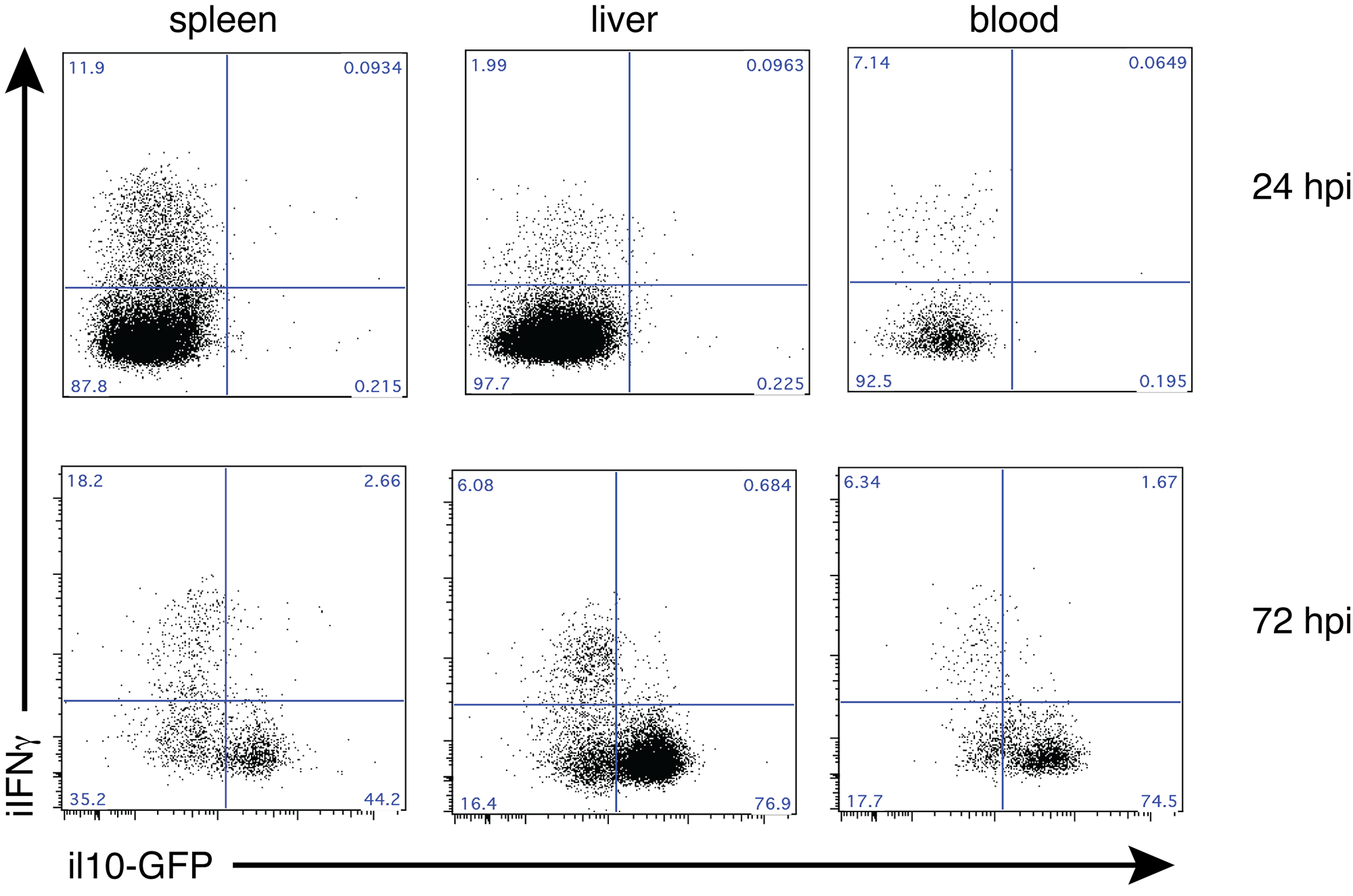

Supplement: S2 Fig — Representative flow cytometry data plots show iIFNγ and il10-gfp staining on CD3-NK1.1+ gated cells isolated from the spleen, liver, and blood of tiger IL-10 GFP-reporter mice at 24 or 72 hpi with 104 Lm. (TIF) [file ppat.1005708.s002.tif]

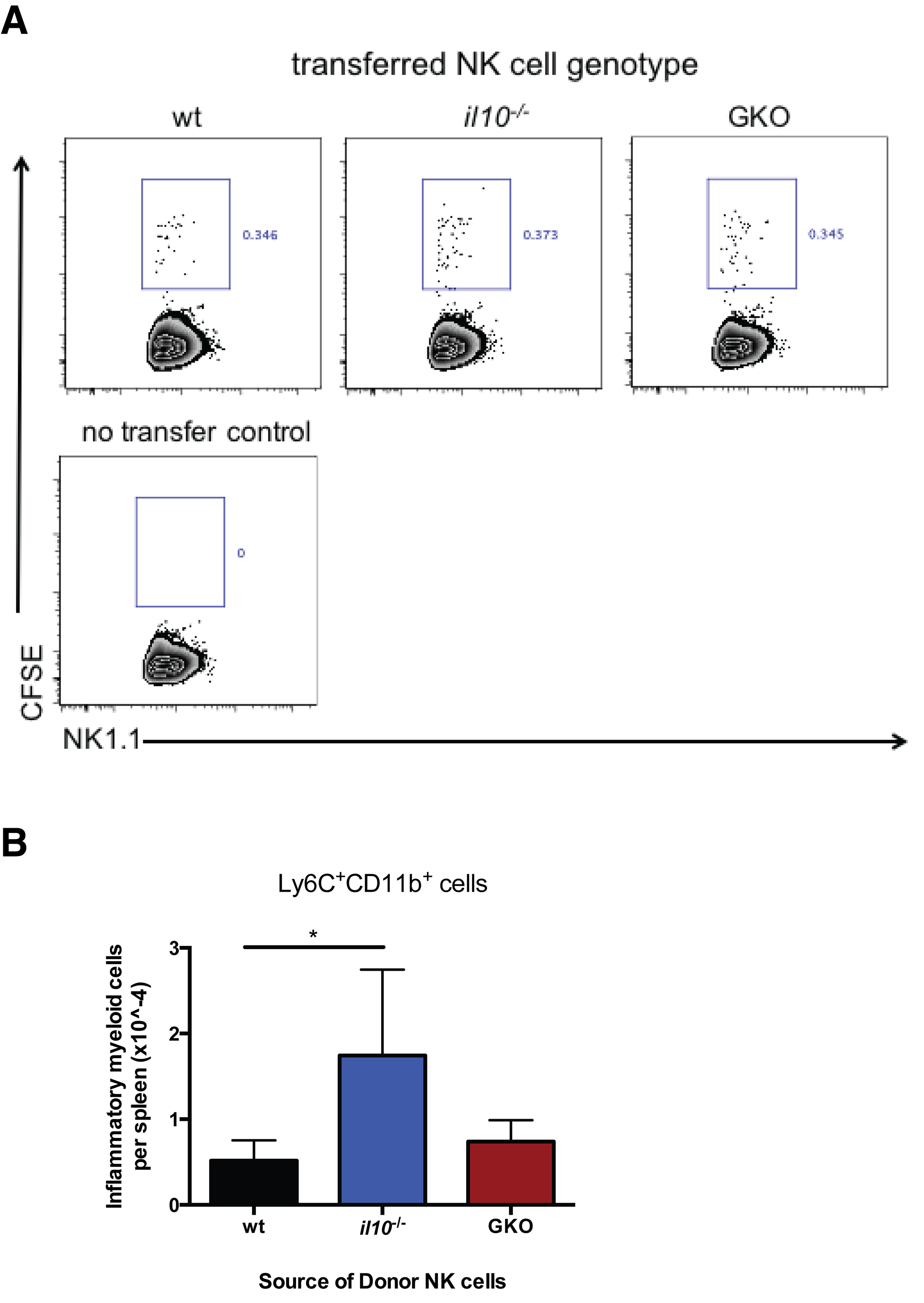

Supplement: S3 Fig — (A) CFSE and NK1.1 staining on CD3-NK1.1+ gated cells isolated from the spleens of IL-10-/- recipient mice at 72 hr post transfer of CFSE-labeled purified NK cells and 96 hpi with 104 Lm. (B) Total numbers of Ly6C+CD11b+ inflammatory myeloid cells per spleen at 96 hpi in IL-10-/- mice that received NK cells purified from wt, il10 -/-, or GKO mice. *P < .05 by ANOVA. (TIF) [file ppat.1005708.s003.tif]

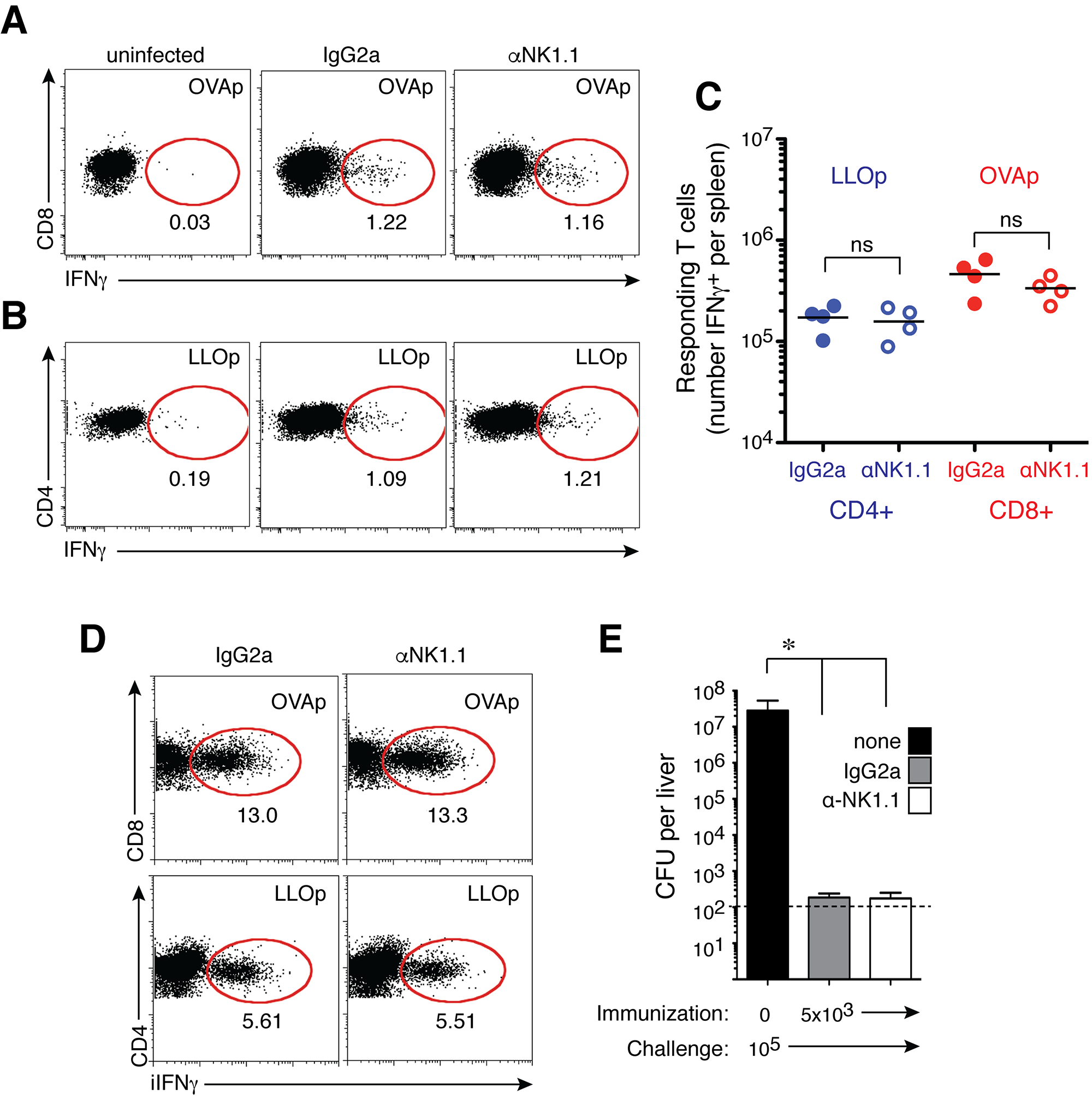

Supplement: S4 Fig — (A-C) Control IgG2a or αNK1.1 Abs were given 24 h before priming of mice with live Lm-OVA (5000 CFU). At 7 dpi splenocytes were harvested and stimulated with synthetic peptides corresponding to Lm-derived epitopes from ovalbumin (OVAp) or LLO (LLOp). (A) Intracellular IFNγ in gated CD3+8+ splenocytes. (B) Intracellular IFNγ in gated CD3+4+ splenocytes. (C) Numbers of responding CD4+ and CD8+ cells per spleen calculated from the respective gated iIFNγ+ populations. Symbols represent values from individual mice and horizontal lines indicate means. Shown are data from one of two experiments using n = 4 mice/group. (D-E) Mice immunized as above were re-challenged using a high dose (105 CFU) of Lm-OVA 28 d later. (D) Intracellular IFNγ in gated CD3+4+ and CD3+8+ T cells from spleens of the re-challenged mice. Staining was done at 96 h after challenge. Plots are representative of 4–5 mice/group. (E) Liver Lm burdens from mice in (D). Age-matched control mice received challenge without prior immunization. *P < .05 by ANOVA. (TIF) [file ppat.1005708.s004.tif]
